# Daily station-level records of air temperature, snow depth, and ground temperature in the Northern Hemisphere

**DOI:** 10.1038/s41597-024-03483-x

**Published:** 2024-06-18

**Authors:** Vinh Ngoc Tran, Wenbo Zhou, Taeho Kim, Valeriy Mazepa, Victor Valdayskikh, Valeriy Y. Ivanov

**Affiliations:** 1 Department of Civil and Environmental Engineering, University of Michigan, Ann Arbor, MI 48109 USA; 2 Institute of Plant and Animal Ecology, the Ural Branch of the Russian Academy of Sciences, Yekaterinburg, Russia; 3 Ural Federal University, Yekaterinburg, Russia

**Keywords:** Atmospheric science, Cryospheric science

## Abstract

Air temperature (Ta), snow depth (Sd), and soil temperature (Tg) are crucial variables for studying the above- and below-ground thermal conditions, especially in high latitudes. However, *in-situ* observations are frequently sparse and inconsistent across various datasets, with a significant amount of missing data. This study has assembled a comprehensive dataset of *in-situ* observations of Ta, Sd, and Tg for the Northern Hemisphere (higher than 30°N latitude), spanning 1960–2021. This dataset encompasses metadata and daily data time series for 27,768, 32,417, and 659 gages for Ta, Sd, and Tg, respectively. Using the ERA5-Land reanalysis data product, we applied deep learning methodology to reconstruct the missing data that account for 54.5%, 59.3%, and 74.3% of Ta, Sd, and Tg daily time series, respectively. The obtained high temporal resolution dataset can be used to better understand physical phenomena and relevant mechanisms, such as the dynamics of land-surface-atmosphere energy exchange, snowpack, and permafrost.

## Background & Summary

Air temperature (Ta), snow cover depth (Sd), and ground temperature (Tg) are essential hydrometeorological states to consider when investigating thermal regimes in the mid- and high-latitudes, such as the Arctic and sub-Arctic regions^1,2^. Here, we define the ground temperature (also referred to as soil temperature) as the subsurface thermal state below the land surface measured at depths ranging from a few centimeters to several hundred meters (monitoring depth range depends on station). Ta, Sd, and Tg have high importance for understanding the impacts of climate on land-surface-atmosphere energy exchange, snow processes, and the permafrost – a sensitive and insufficiently monitored component of the planet’s cryosphere^1,3–5^. To address relevant science questions, changes in the air and ground thermal states and snow cover depth need to be observed^2^. Changing vegetation distribution and animal habitats in the mid- and high-latitude regions are also tied to these thermal and snow variables^6–9^.

In order to assess the regional hydrometeorological and ground thermal regimes more accurately, particularly in terms of their extremes, comprehensive observational datasets on *daily* Ta, Sd, and Tg are required^10–14^. Compilations of such datasets to cover large spatial domains are still insufficient. Studies that use observed daily data are usually confined to small spatial scales^15–18^, while those conducted on a broader scale predominately consider data of coarser, annual temporal resolutions^16,19,20^ or reanalysis data products^21–24^.

Specifically, while there is an abundance of datasets providing data on Ta, Sd, and/or Tg^12,25–27^, their temporal characteristics can be highly inconsistent (e.g., a station can have time-varying temporal resolutions) and contain numerous missing data points. Such datasets also mix multiple resolutions, such as sub-daily, daily, weekly, monthly, and yearly. Furthermore, though reanalysis data products, such as ERA5^28^ and ERA5-Land^29^ offer contiguous climate variables at a high temporal resolution, the spatial resolution provided by such models can be coarse and unable to provide the detail necessary to capture local-scale variations that can be represented by *in-situ* observations. Remote sensing-based data products on air temperature^30,31^ and snow depth^32,33^ have been increasingly used over the past decade. But they are often fraught with substantial uncertainties that require complex post-processing and validation against ground-based data to be used with confidence^30,34,35^. In addition, some datasets were created by integrating various data-sources (e.g., satellite images, reanalysis data, and ground-based data), but only for specific areas or countries, particularly Ta^36–39^ and Sd^40–44^. Given an extensive range of Ta, Sd, and Tg potential applications, the above considerations encourage the need for a uniform, standardized daily dataset of these variables at a large scale.

In recent years, the proliferation of machine learning (ML) and deep learning (DL) has enabled researchers to tackle more challenging problems of reconstructing and deriving complex and diverse types of data^45–47^. These algorithms do not rely on predetermined equations and assumptions, such as traditional process-based or mathematical models do, but rather adapt and learn from data to identify hidden patterns and underlying correlations^48^. This makes ML/DL an attractive option for researchers seeking to attain superior accuracy in data estimation. Numerous studies have already demonstrated the efficacy of ML/DL tools for reconstructing missing data on Ta, Sd, and Tg in spatially small regions^31,49–55^.

In this research, we aim to derive a consistent, station-based dataset on *daily* Ta (2 m above the land surface), Sd, and Tg (at different soil depths). Specifically, the geographic scope encompasses the broader Northern Hemisphere north of 30°N latitude, including the data-sparse Arctic region, with the goal of enabling diverse applications of the resultant dataset. The reason for choosing a 30°N latitude as the southern limit is that there are no stations that have measured ground temperature below this latitude in the Northern Hemisphere. This selection does not affect our goal of reconstructing a data set that has a focus on the Arctic and pan-Arctic regions. We integrated various data sources in which the missing values were derived through DL that relied on a reanalysis data product. We developed a comprehensive, observed-reconstructed database of Ta, Sd, and Tg for the period of 1960–2021. This database can be used to analyse local-scale dynamics and relationships between Ta, Sd, and Tg to help refine earth-system models simulating land-surface-atmosphere heat exchange, snow processes, and trends in the permafrost state. Furthermore, the derived dataset can be utilized to improve the accuracy of global gridded reanalysis or satellite-based products.

## Methods

### Data acquisition

**Fig. 1:**
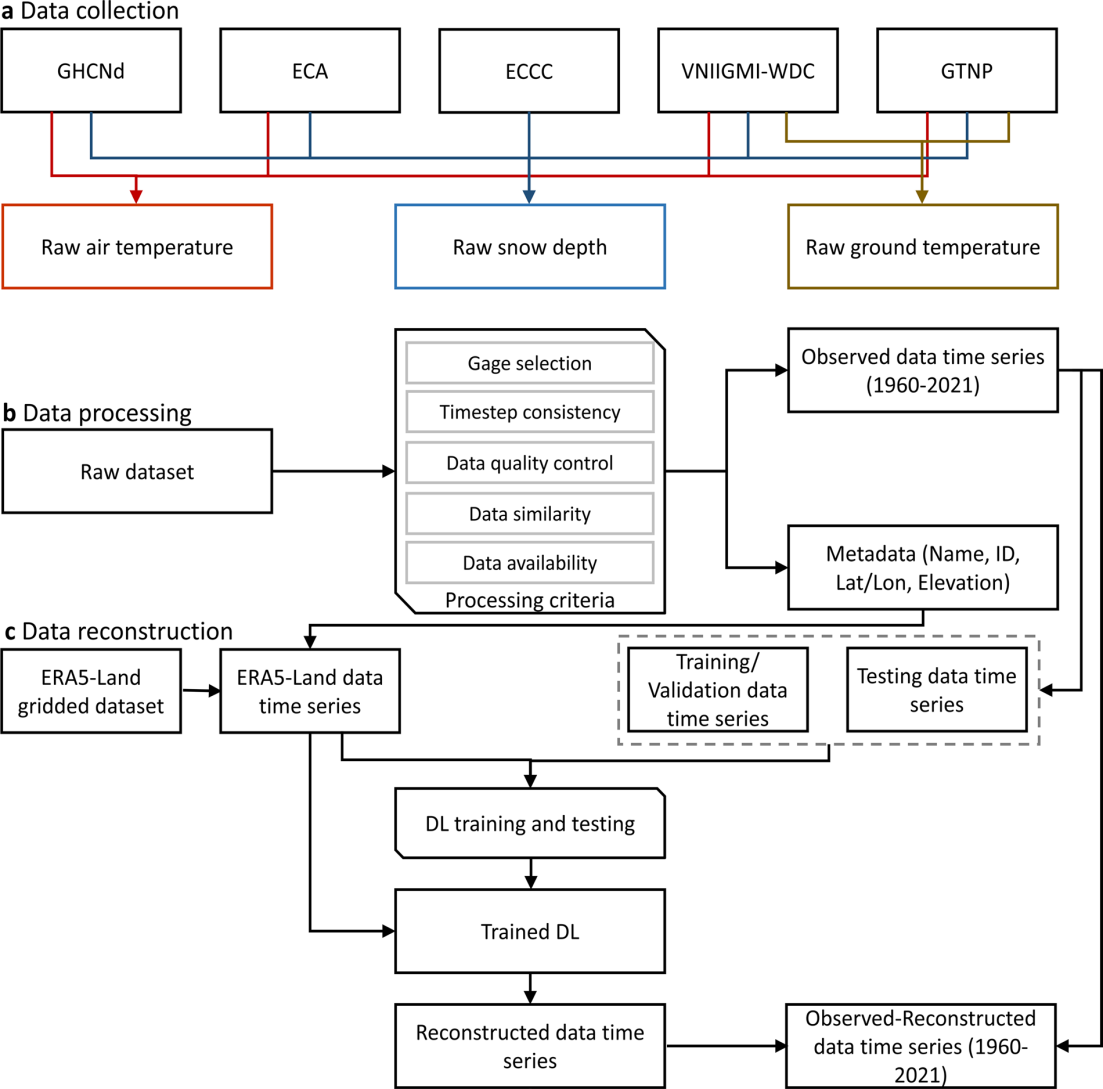
A schematic overview of the data reconstruction workflow. Part (**a**) illustrates the collection of raw (or observed) data on air temperature (Ta), snow depth (Sd), and ground temperature (Tg) from six distinct sources, which include the Global Historical Climatology Network daily (GHCNd), the European Climate Assessment & Dataset (ECA), the Environment and Climate Change Canada (ECCC), the All-Russian Research Institute of Hydrometeorological Information-the World Data Center (VNIIGMI-WDC), and the Global Terrestrial Network for Permafrost (GTNP). Part (**b**) shows the data processing workflow to develop daily data series for all locations over a consistent time frame spanning 1960/01/01 - 2021/12/31. The data set also includes metadata specifying the station’s name, identification number (ID), coordinates (lat/lon), and elevation above sea level. Part (**c**) outlines reconstruction of the missing data using deep learning (DL) and reanalysis data product (ERA5-Land). Observations are used for training, validating, and testing the DL model. The reconstructed data are then combined with the observed data to provide a complete observed-derived data series.

The implementation procedure used to generate the dataset is displayed in Fig. 1. Initially, we integrated a significant amount of data from five distinct datasets from different sources. The collected data contained time series of *in-situ* measurements. We only used observational datasets that were publicly available (Fig. 1a).

Specifically, the first dataset used for this study is the Global Historical Climatology Network - Daily (GHCNd)^25^, which is comprised of the daily data series for a total of 123,176 stations with various climate variables, including Ta and Sd. Next, the European Climate Assessment & Dataset (ECA)^12^ was accessed that contains Ta and Sd data for 6,628 and 9,798 sites, respectively. Third, Environment and Climate Change Canada (ECCC)^56,57^ provided a dataset consisting of 5,719 locations with Sd records. The fourth repository downloaded from the All-Russian Research Institute of Hydrometeorological Information - the World Data Center (VNIIGMI-WDC) (http://nodc.meteo.ru/) offered 600 sites for Ta, 620 sites for Sd, and 459 sites with records on Tg. Lastly, the dataset by the Global Terrestrial Network for Permafrost (GTNP)^58^ that has been used in numerous studies on ground thermal regime was also integrated and it contained a total of 1,385 locations. Note that air temperature for all datasets was observed at 2 m above the land surface.

The ERA5-Land reanalysis dataset, sourced from the European Centre for Medium-Range Weather Forecasts (ECMWF), was used to derive the missing data for all three variables (Ta, Sd, and Tg). The variables from ERA5-Land were selected based on the prior analogous studies that carried out data reconstruction for Ta, Sd, and Tg^31,49–55^. Specifically, the utilized variables are air and dewpoint temperatures at 2 m above ground, soil type, soil temperature levels 1 (0–7 cm) to 4 (100–289 cm), snow depth, snowfall, surface latent and sensible heat fluxes, surface net solar and thermal radiations, and surface pressure. The dataset encompasses the time period from 1960 to 2021 at a 0.1-degree spatial resolution.

There are several reasons behind the selection of ERA5-Land dataset for this reconstruction. First, the accuracy of this data product has been enhanced by assimilation of observed data, making it a primary choice for analysis by researchers who used ERA5 or ERA5-Land in various previous investigations^29,59–62^. Second, this dataset offers a highly detailed spatial (0.1 degrees) and temporal (1-hour) resolutions on a global scale, that also correspond to a long-term period of more than 70 years (starting from 1950). Third, ERA5 data product provides a suit of climate and ground surface variables (such as air temperature, precipitation, wind speed, soil moisture, among others), making it suitable for this research aimed at deriving missing data for Ta, Sd, and Tg.

### Data processing

The observed data were filtered, standardized into consistent daily time series, and consolidated to eliminate partial duplicates (Fig. 1b). The standardized dataset includes the time series of Ta, Sd, and Tg, dating as early as 1960/01/01 and as late 2021/12/31. Observations are accompanied by metadata that include basic station information such as name, identification number (ID), geographic coordinates (latitude/longitude), and elevation. The specific steps and criteria used in the data processing are presented as follows.

- *Station selection:* We chose a set of monitoring stations contained within the Northern Hemisphere that are situated north of 30 degrees latitude.

1 $$Latitude\ge 3{0}^{o}$$

- *Timestep consistency:* Given the heterogeneity in the temporal resolution of the five observational datasets, we adopted a filtering process to identify records that have data available at temporal resolutions ranging from hourly to daily. The time series that originally were at an hourly or sub-daily resolution were averaged to convert them into daily data series. The units for Ta, Sd, and Tg were standardised to Celsius degree, meter, and Celsius degree, respectively. Finally, the metadata associated with the identified stations were compiled based on their original record.
- *Data quality control:* It should be noted that not all observational data can be unconditionally trusted, as measurement errors can introduce inaccuracies. Ideally, all selected data sets should be accompanied with a confidence assessment of each daily value. In this study, most utilized datasets assign quality flags to each value (as shown in Table 1), except for the GTNP dataset. For these flagged datasets, only values that appear as ‘valid’ or ‘did not fail any quality control checks’ were accepted during the processing. Conversely, any data identified as ‘suspect’, ‘incorrect’ or ‘failing quality control tests’ were discarded. Since the GTNP dataset was already thoroughly evaluated and filtered in previous research^58^, it was reasonable to assume all recorded data are valid.

**Table 1:** Quality flags of observations in the collected datasets.

Dataset	Quality code
GHCNd	Blank: missing data
	Quality flags were provided for each data point:
	Blank: did not fail any quality assurance check
	D, G, I, K, L, M, N, O, R, S, T, W, X, Z: failed quality check
ECA	Quality flags were provided for each data point: 0: Valid data; 1: suspect data; 9: missing data
ECCC	Quality flags were provided for each data point:
	0, 10, 20: Valid data
	1, 11, 21: Today’s snow depth is greater than red flag maximum value (300 cm)
	2, 12, 22: No change or increase in snow depth when melt expected (Tmax> 4.5 °C)
	3, 13, 23: No change or decrease in snow depth when accumulation expected (Tmax <−1 °C and snowfall> = 5 cm)
	4, 14, 24: Snow depth increased by more than 3 cm with no snowfall
	5, 15, 25: Snow depth decreased by more than 3 cm under freezing conditions (Tmax < −1 °C) with snowfall (> = 5 cm)
	6, 16, 26: Snow depth decreased by >10 cm under non-rain conditions and air temperatures <4.5 °C
	7, 17, 27: Snow depth reduction exceeds red flag condition (30 cm)
	8, 18, 28: QC impossible due to missing data
	9, 19, 29: QC impossible due to missing data and snow depth >300 cm
	100: Missing snow depths determined to be zero values
	101–365: Gap filled estimate where the closest observation is flag-100 days away. Gap-filled values within 7 days of an observation were found to introduce only small errors into snow depth time series by Brown and Braaten (1998)
	467: Values entirely reconstructed from daily climate data
	999: Missing value
VNIIGMI-WDC	Quality flags were provided for each data point:
	For Ta: 0: Valid data; 1: Incorrect data; 9: Missing data
	For Sd: 0: Valid data; 1: No snow
	2: No snow cover at the gage, but there is snow in the nearby area
	3: Snow depth <0.5 cm; 9: Missing data
	For Tg: 0: Valid data; 1–4, 8: Incorrect data; 5–7: Suspect data; 9: Missing data
GPNT	−999: Missing data
	Quality flag was not provided

2 $$Flag={\rm{valid}}| {\rm{did}}\;{\rm{not}}\;{\rm{fail}}\;{\rm{any}}\;{\rm{quality}}\;{\rm{assurance}}\;{\rm{check}}$$

- *Data similarity:* By considering both geospatial and temporal information, we were able to identify overlapping datasets/gages. Specifically, to accomplish this, we assessed the data by evaluating station geographic proximity and the time series correlation between different stations in order to identify duplicates and subsequently identify duplicate time series.
  In cases where two stations have inconsistent temporal data coverage, only information such as coordinates, gage names, IDs, and elevations were used to identify potential duplicates. Specifically, the conditions that need to be satisfied, as in Eq. (3), are as follows: (1) the distance (*d*_*a-b*_) between the two stations (e.g., *a* and *b*) must be less than 1 km; (2) the elevation difference between the two stations (*h*_*a-b*_ should not exceed 1 meter; and (3) the names or IDs of the two gages must match.
  3 $$\left\{\begin{array}{c}{d}_{a-b} < 1km\\ \begin{array}{c}{h}_{a-b} < 1m\\ Nam{e}_{a}\equiv Nam{e}_{b}| I{D}_{a}\equiv I{D}_{b}\end{array}\end{array}\right.$$
  For stations with temporally consistent data coverage, in addition to geographic proximity and elevation similarity, the correlation between the two data series was evaluated to determine duplicity. Specifically, we calculated the Pearson Correlation coefficient (*R*_*a-b*_) between respective time series and used the criterion of *R*_*a-b*_ > 0.99 to determine whether the two time series were “very likely identical”, following the approach used in previous studies^63,64^. For stations that satisfied the criteria of Eqs. (3, 4), the data were merged when possible, with overlapping data segments that were subjected to averaging, so that a data series of longer duration can be produced. In the cases where duplicate gages were identified with inconsistencies in metadata such as coordinates, names, IDs, and elevations, the metadata were selected based on the dataset precedence order presented in Table 1.
  4 $$\left\{\begin{array}{c}{d}_{a-b} < 1km\\ \begin{array}{c}{h}_{a-b} < 1m\\ {R}_{a-b} > 0.99\end{array}\end{array}\right.$$
- *Data availability:* Stations that have the number of available records (*N*_*data*_) (not necessarily consecutive) greater than 365 days over the period 1960 to 2021, their record is retained if:

5 $${N}_{data}\ge 365$$

The records were discarded otherwise. The rationale for selecting data series exceeding 365 days stemmed from the assumption that a full year’s worth of data would offer adequate temporal resolution and capture the seasonal variations in the target variables (Ta, Sd, and Tg), potentially aiding the ML model in identifying these periodicities in the predictor variables.

### Reconstruction of the missing data

The missing data are inferred through the use of a DL model (Fig. 1c). To achieve this, we employ a Long Short-Term Memory (LSTM) with attention mechanism (attention-based LSTM) to train reconstruction models. LSTM is a special kind of recurrent neural network (RNN) that is focused on resolving the vanishing gradient problem^65^ that traditional RNNs frequently encounter, while attempting to capture long-term dependencies across successive data periods^66^. An internal memory cell enables LSTMs to maintain and recall information across extended sequences, allowing it to perform tasks involving time series data^67^. These memory cells are modulated by three gates – input, forget and output – enabling LSTMs to appropriately store, update, and retrieve the data information. The attention mechanism assigns scores to each input feature, thereby enabling the LSTM network to consider the interdependence of input sequences at different temporal steps in detail^68^, improving the ability of LSTM to capture nonlinear dependencies^69–71^. In addition, the attention-based LSTM also has the benefit of eliminating the need for manual selection of appropriate input predictors, since all the candidate input predictors can be used simultaneously to predict the target outputs and the LSTM can self-determine the relative importance of each input factor by assigning attention weights to them.

In this work, we perform station-based reconstruction rather than gridded data reconstruction. That is, an individual LSTM was trained to reconstruct data for a specific station. Missing data at each station are reconstructed using LSTM. For Tg data, only the depths with observed data are reconstructed, so the vertical resolution of Tg is not equal for all stations. The data preparation for a DL model begins with an extraction of data series of input predictors, including days of the year time series (the values ranging from 1 to 366) and data from the ERA5-Land gridded dataset for the locations of Ta, Sd, and Tg that have missing data. ERA5-Land dataset contains the time series of multiple hydrometeorological variables such as 2 m air temperature, 2 m dewpoint temperature, soil type, soil temperature profile (levels 1 to 4), snow depth, snowfall, latent and sensible heat fluxes on the surface, surface net thermal radiation, and surface pressure. The period of ERA5-Land dataset extends beyond the period of 1960 to 2021, the timespan of the reconstructed time series.

Ta, Sd, and Tg are used as DL target variables. The data series for each variable from 1960 to 2021 is partitioned into two subsets. The first subset is comprised of available data (along with their corresponding timestamps) and used for training, evaluating, and testing the model. In this subset, 80% of the available data is allocated for training and evaluating the model, labelled as the ‘training/validation set’. The remaining 20% of the data is used for testing the model, termed the ‘testing set’. The second subset is composed of timestamps of missing data, termed the “missing timestamp”. Subsequent to the inspection of the timestamps of all of the data sets, the input predictor data series are extracted from ERA5-Land. The dataset comprises input predictor data sequences with missing timestamps termed the “application set”. Before feeding the data into the LSTM model for training and application, all data are normalized using the min-max normalization^72^.

DL model configuration required tuning of its hyper-parameters, such as the number of hidden layers, the number of hidden units, the dropout rate, and the batch size^73,74^. This was done using the Bayesian optimization with Gaussian processes, a widely-utilized and highly-efficient approach^75^. We set the value ranges of the DL model four hyper-parameters to [1–2], [64–512], [0–0.9], and [8–512], respectively. The hidden states were initialized with zeros, as they are the default states utilized in Tensorflow^76^. The mean square error was adopted as the loss function, and the ADAM optimizer (with the learning rate of 0.0001) was opted to enhance the training of the model^77^. To optimize effectiveness, an adaptive early stopping technique^78^ was implemented. This technique was designed to early terminate the process when no further improvement in the model’s performance is observed^79^. The maximum number of epochs was predetermined to be 500 for all case study locations.

Once the reconstruction (DL) model has been trained, the “applying set” is used to derive the missing data. These reconstructed data are then combined with the original data to create a complete, continuous data series from 1960 to 2021.

## Data Records

The dataset is available through a Figshare repository^80^. The dataset consists of three compressed files (0.7z), each containing data in text format, and three metadata files for the three variables (Ta, Sd, and Tg). The data for each station is stored in separate text files, and they all have consistent time series from 1960/01/01 to 2021/12/31. The total number of files corresponding to the number of stations provided for Ta, Sd, and Tg are as follows: 27,768 files for Ta, 32,417 files for Sd, and 659 files for Tg. Basic information about the monitoring station, such as the name, identification number (ID), coordinates, gage elevation, and evaluation results, is provided in the metadata files. Evaluation results include values of four metrics for evaluating LSTM performance, including R^2^ (coefficient of determination), KGE (Kling-Gupta efficiency coefficient)^81^, RMSE (root mean square error), and ME (mean error).

For Tg metadata, information about the depths with measured data is also provided. Furthermore, we also provide the results of LSTM for the testing set (stored in the format of a *.mat file) for all case studies so that users can utilize them as a reference or benchmarking study in similar research on data reconstruction.

The format of each file for Ta and Sd variables is consistent, with the columns called “Date,” “Values,” and “Flags.” In this format, the “Values” column includes both observed data and derived data, which are determined by flag values. For example, 1 represents the observed data, and 2 represents the derived data. For Tg data, the columns include “Date,” “Depth_<soil depth>,” and “Flag_<soil depth>.” Since each Tg measurement station has multiple measurement depths, data and flags are provided separately for each depth. The filename for each measurement station is set as “<ID><Latitude><Longitude>.txt,” where latitude and longitude values are rounded to three digits after the decimal point.

## Technical Validation

### Data summary

**Fig. 2:**
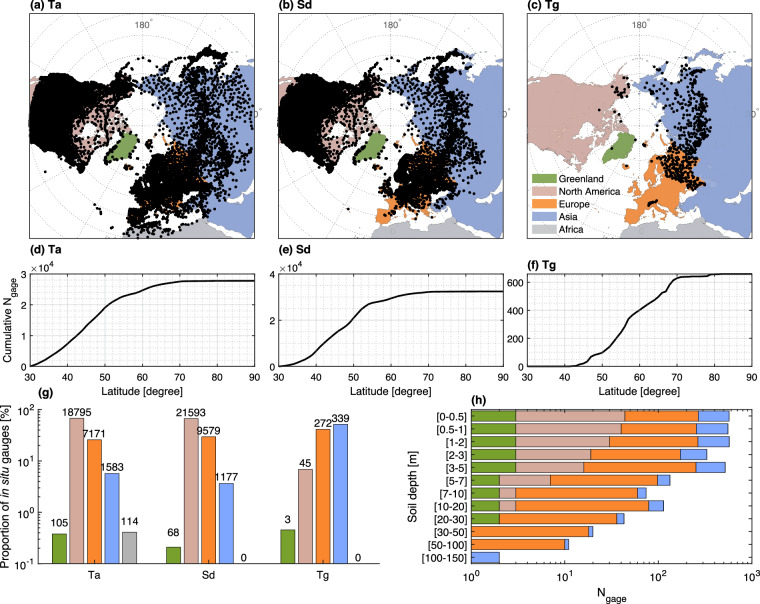
Locations and regional contributions of *in situ* observational records used in this study. Subplots (**a–c**) depict the locations (black dots) of 27,768 air temperature (Ta) stations, 32,417 snow depth (Sd) measurement sites, and 659 locations with ground temperature (Tg) observations that remained after the data processing/filtering. Subplots (**d**–**f**) respectively show the cumulative number of Ta, Sd, and Tg gages counted from 30°N to 90°N. Subplot (**g**) illustrates the corresponding numbers of observational sites for five geographic regions above 30-degree northern latitude, including Greenland, North America, Europe, Asia, and Africa (distinguished with different colors in the subplots). Subplot (**h**) presents the number of Tg measurement locations across the five regions with varying soil depths to 150 meters. Y-axis in (**g**) and x-axis in (**h**) use the log-scale.

**Fig. 3:**
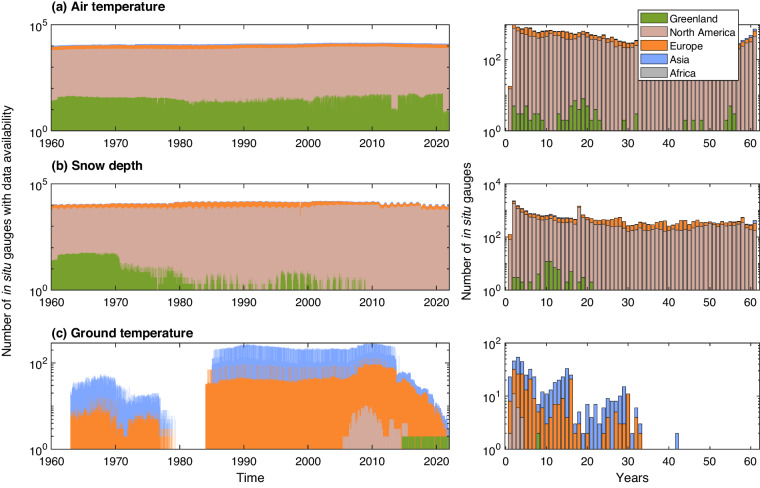
Illustration of the data availability of Ta, Sd, and Tg. The subplots on the left side show the number of stations (y-axis) with available daily data over the period of 1960 to 2021 (x-axis). Subplot (c) illustrates the number of Tg stations that have observed data at all their measured soil depths (note stations may monitor Tg at different soil depths). The right-column subplots show the total number of stations (y-axis) as a function of their record duration (i.e., the cumulative total number of years over which data have been collected) (x-axis). The individual colors of the bar plots represent the number of stations located in each geographic region. The y-axis in all subplots uses the log-scale.

As the original datasets include quality flags and have been verified, we simply collect the data and perform data processing as described in the Methods section. The locations of stations selected after processing are illustrated in Figs. 2, 3. The Ta and Sd gages are broadly spread in five geographic regions (Greenland, North America, Europe, Asia and Africa). One may note that Ta and Sd measurement locations are abundantly present in North America, representing over 60% of all stations, while Europe is the second most represented region, hosting additional 25.8% (Ta) and 29.5% (Sd) stations. Sites of Tg observations are mainly located in Asia (51.4%), followed by Europe (41.3%). The southernmost continent Africa has the fewest number of stations, with only Ta data available for 114 stations (about 0.004%).

The number of Tg monitoring sites as well as the observed soil depths are shown in Fig. 2h. It can be inferred that the measurement sites primarily provide Tg at depths ranging from 0 to 5 meters. Sites in Europe and a few sites in Asia provide measurements at greater depths, ranging from 50 to 150 meters. Specifically, it can be observed that there are more stations in Europe with data collected at depths of 5 to 100 meters as compared to the other regions. At soil depths greater than 100 meters, only Asia has stations with data, with a total of 2 stations.

Examination of the data revealed substantial amounts of missing values for the observed Ta, Sd, and Tg, with 54.5%, 59.3%, and 74.3% of the data missing for the considered period. Figure 3 provides an overview of available data from 1960 to 2021. The number of stations with available data fluctuates over time (left subplots of Fig. 3) and the total record duration varies widely across stations (right subplots of Fig. 3). Specifically, 51.3% of Ta stations have just 1 to 20 years of observations, while only 4.7% have records exceeding 60 years. The snow depth data exhibit a similar pattern, with very few stations that have records exceeding 60 years, that only accounts for 2.2% of the 32,417 stations. Notably, 2,271 Sd stations (7.2%) have data spanning merely 2 years.

Figure 3c presents statistics for stations with available Tg data across all their measured soil depths (note that stations may monitor Tg at different depths). It is apparent that during the period between 1960 and 1983 and after 2014, there were few locations with extensive data. A considerable proportion of sites have data sets limited to durations of 1–17 years, while a limited number of stations have the total number of records reaching 40 years (Fig. 3c-right plot).

Evidently, such widespread absence of measurements precludes robust quantification of long-term trends, with the large fractions of missing data undermining capacity to characterize baseline climate, variability, or detection of changes for these variables. The considerable spatiotemporal deficiencies and discontinuities within the terrestrial observation network pose significant challenges for resolving historical patterns or recent trajectory of warming, snow cover fluctuations, and subsurface thermal shifts across the analysis domain. Fundamentally, analyses aimed at detecting tendencies in climate variables depend on extensive, uninterrupted records^82–85^. This further highlights the need for continuous reconstructed data series.

### Validation of derived data

The performance of the trained DL model in deriving Ta, Sd, and Tg data is assessed using a testing set representing 20% of the total observed data with respect to each gage. To evaluate the model’s efficacy, two common metrics are used. Specifically, they are the R^2^, KGE, RMSE, and ME. These metrics are commonly used to evaluate the skill of models yielding time series. The range of values for R^2^, KGE, RMSE, and ME is [0, 1] (-∞, 1], [0, +∞], and (-∞, +∞] respectively, with values of 1, 1, 0, and 0 indicating ideal performance where the derived data perfectly match the observed data. Here, we primarily rely on the R^2^ and KGE metrics to evaluate the performance of the LSTM. RMSE and ME results are presented in Supplementary Figs. 1, 2. and within the publicly shared dataset to provide additional quantitative evidence regarding reconstruction fidelity. The model’s results are considered satisfactory when the values of R^2^ and KGE are greater than thresholds of 0.5 and 0.3^86^, respectively. Since R^2^ is a well-known coefficient, only KGE formula is illustrated, as in Eq. (6).

6 $${\rm{KGE}}=1-\sqrt{{\left({\rm{r}}-1\right)}^{2}+{\left(\frac{{{\rm{\sigma }}}_{{\rm{Der}}}}{{{\rm{\sigma }}}_{{\rm{Obs}}}}-1\right)}^{2}+{\left(\frac{{{\rm{\mu }}}_{{\rm{Der}}}}{{{\rm{\mu }}}_{{\rm{Obs}}}}-1\right)}^{2}}$$

where r is the linear correlation between the derived (Der) and observed data (Obs); σ_Obs_ and σ_Der_ are the standard deviations of Obs and Der, respectively; μ_Obs_ and μ_Der_ are the means of Obs and Der, respectively.

**Fig. 4:**
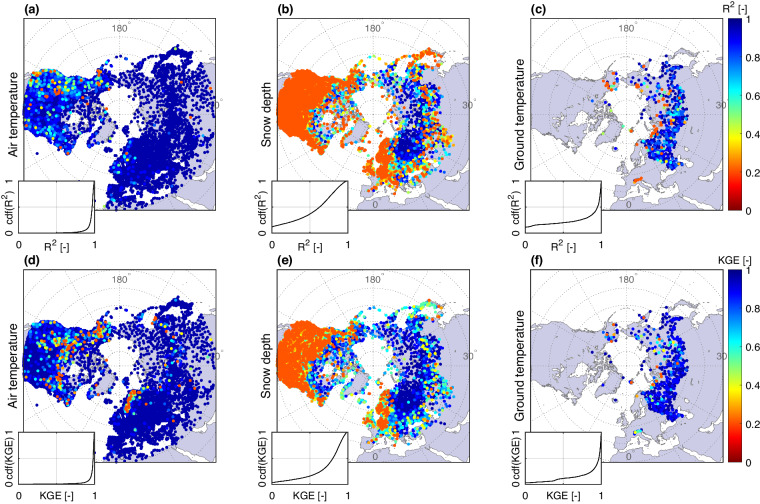
The performance of the DL model in deriving the data using the testing set on air temperature (Ta) (**a,d**), snow depth (Sd) (**b,e**), and ground temperature (Tg) (**c,f**). The performance is quantified using the coefficient of determination (R^2^) (a-c subplots) and Kling-Gupta efficiency (KGE) (d-f subplots). The calculated values of R^2^ and KGE for Tg are obtained by averaging these performance metrics across all soil depths for each location. The insets illustrate cdf(R^2^) and cdf(KGE) that are the cumulative distribution functions (cdf) for R^2^ and KGE, respectively. Poorly performing stations (represented by red dots) are highlighted by placing their markers on top of symbols for adequately performing stations. The locations of stations with low R2 and KGE values are prioritized for plotting over the locations of stations with higher accuracy.

**Fig. 5:**
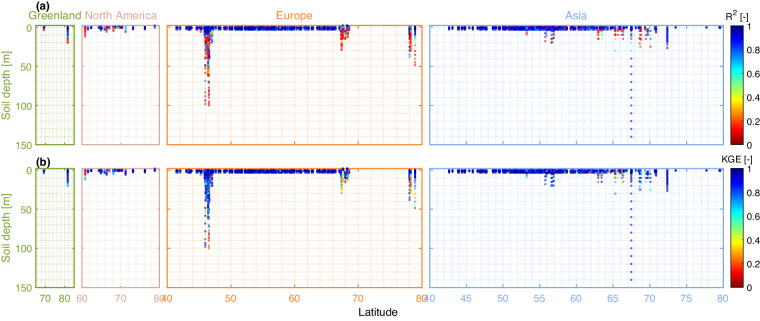
The DL performance in estimating observations using the testing set on Tg for each location and soil depth. The performance is quantified using R^2^ (the upper panel) and KGE (the lower panel). The subplots are for four geographic regions: Greenland, North America, Europe, and Asia (note that there are no available Tg records in Africa). Poorly performing stations (represented by red dots) are highlighted by placing their markers on top of symbols for adequately performing stations. The locations and soil depths of stations with low R2 and KGE values are prioritized for plotting over the locations and soil depths of stations with higher accuracy.

**Fig. 6:**
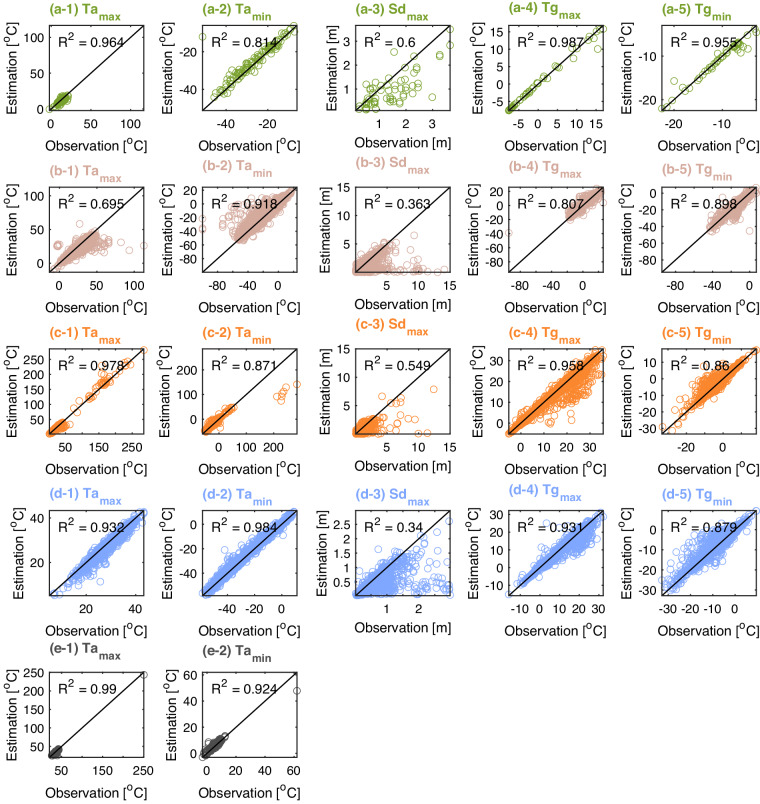
Comparisons of observations and derived values for the testing set for five different geographical regions (a-Greenland, b-North America, c-Europe, d-Asia, and e-Africa). The 1:1 comparisons are presented in subplots (**a**–**o**)) using maximum and minimum air temperature (Ta_max_ and Ta_min_), maximum snow depth (Sd_max_), and the maximum and minimum ground temperatures at every soil depths (Tg_max_ and Tg_min_). The color-coding of each subplot corresponds to the region colors used in Fig. 2.

**Fig. 7:**
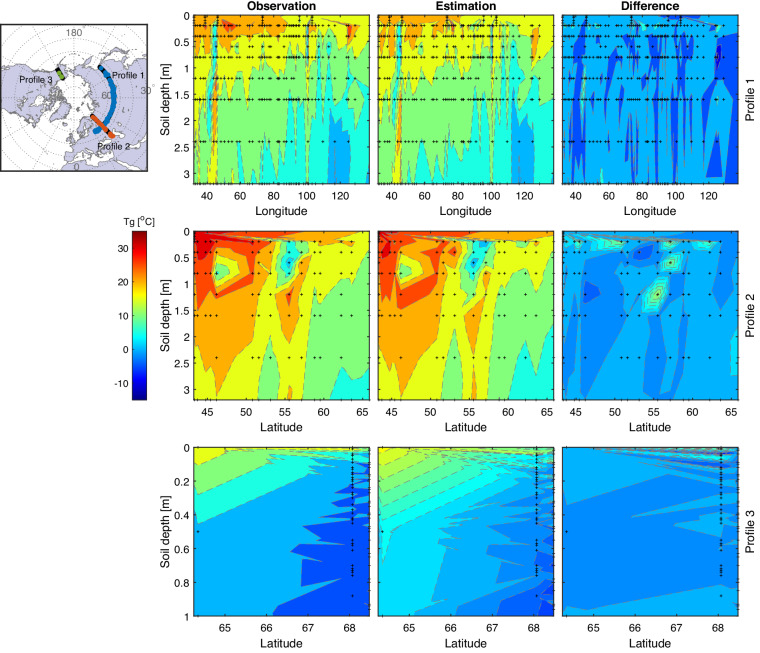
An illustration of maximum ground temperatures with depth for the testing set along the three profiles shown in Supplementary Fig. 1. The left set of plots (“Observation”) displays the observed data used in the testing set. The middle set (“Derivation”) shows the reconstructed data using the DL model. The right set of plots shows the difference between the data shown in the first two columns (“Difference” = “Observation” – “Estimation”). The small black dots indicate the longitudinal/latitudinal locations and soil depths of the Tg stations in the three profiles (top to bottom). The distributions of Tg are displayed using the contour plots that linearly interpolate temperature values at the station locations. The map in the upper left shows the three profiles and their nearby ground stations (within 100 km radius from each profile, as circles).

**Fig. 8:**
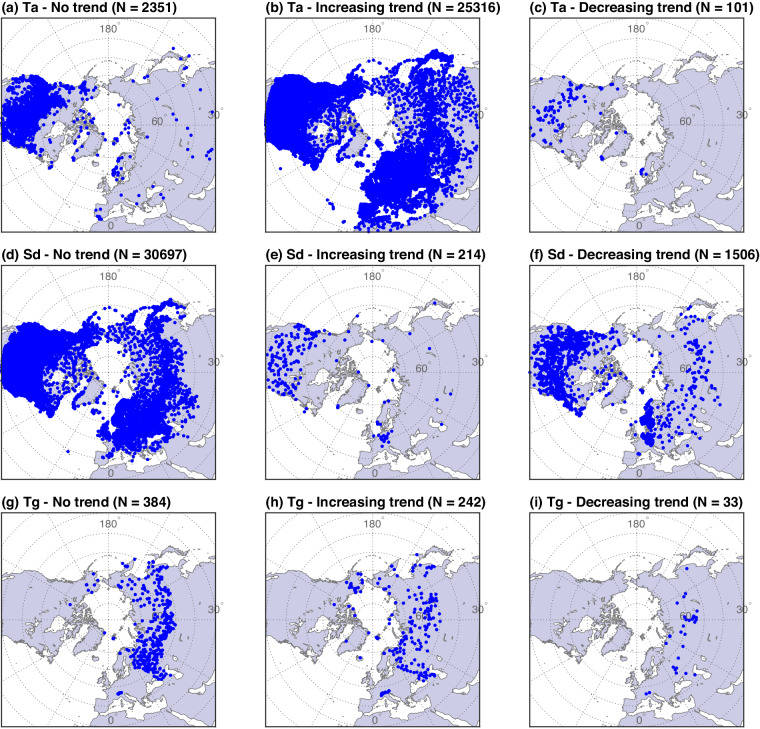
The results of trend analysis using the reconstructed data for Ta, Sd, and Tg from 1960 to 2021. The subplots in the left column show the locations of stations where the reconstructed time series do not reveal a clear trend. The subplots in the middle and right columns show the locations of stations with increasing and decreasing trends, respectively. The number of measurement stations (N) is shown in each subplot title.

Validation results for the LSTM model are shown in Figs. 4–8. It can be seen that the R^2^ and KGE values are relatively high, with most values exceeding 0.5, which signifies satisfactory performance of the trained DL models. Particularly, Ta results were notably high, as R^2^ and KGE values usually exceed 0.7 for most stations (accounting for 97.2%). With regards to Sd, 20,489 (63.2%) sites met the satisfactory threshold for R^2^ > 0.5 and 29,181 (90%) sites with KGE > 0.3.

The LSTM’s performance in deriving Tg is almost similar, with approximately 75% and 95% of the gages meeting the R^2^ and KGE thresholds (i.e., R^2^ > 0.5 and KGE > 0.3). It also can be observed that the performance of the LSTM decreases with increasing depth, especially for stations in the European region (Fig. 5). In general, the LSTM model performs well at depths ranging within 0–5 meters. At greater depths, typically beyond 10 meters, ground temperature is considered to be less influenced by external factors such as surface/air temperature^87,88^. Thus, the performance of the LSTM appears to be lower and less stable at these larger depths. Note that the ERA5-Land dataset only provides soil temperature estimates down to a maximum depth of 2.89 meters. Additionally, the evaluation results show that the reconstruction of Tg is more biased compared to the reconstruction of Ta. Specifically, the subplots of the cumulative distribution function (cdf) in Supplementary Fig. 1a,c demonstrate that the proportion of stations with RMSE greater than 2.3 °C is considerably higher for Tg than the corresponding proportion for Ta. This is understandable as reconstructing Tg is more challenging than Ta, not only because the length of the Tg data series used to train the model is shorter, but also because the relationship between Tg and the input variables is more complex than that of Ta.

The analysis of extreme values (both low and high) is crucial in datasets such as Ta, Sd, and Tg. In this study, five statistical metrics were calculated from the testing set for each station, including maximum and minimum Ta (Ta_max_ and Ta_min_), maximum Sd (Sd_max_), and maximum and minimum Tg (Tg_max_ and Tg_min_). The 1:1 comparisons between the reconstructed and observed data for 5 geographic regions are shown in Fig. 6. The overall comparison results indicate that LSTM is capable of providing relatively good results for extreme values, especially for Ta and Tg, with most R^2^ being above 0.5. Specifically, Ta_max_ and Ta_min_ displayed remarkable correlation with R^2^ of over 0.68 and 0.82 respectively. A similar outcome can be seen for Tg, with the reconstructed data showing R^2^ greater than 0.8. The performance of results calculating Sd_max_ is somewhat lower. Specifically, the results for Greenland, North America, and Europe demonstrate good performance of the trained models with R^2^ values of 0.6, 0.864, and 0.549, respectively. Results for the Asia region show lower skill, with an R^2^ of 0.34.

Figure 7 depicts the performance of ML in deriving Tg_max_ for the testing set at various soil depths along three cross-sectional profiles. Specifically, these profiles were selected based on the density of gages in the regions with the highest number of gages for Tg, with one profile passing through the European-Asian region (longitudinal), one through Europe (latitudinal), and one in Alaska (latitudinal). Tg sites located within 100 km radius from these profiles were used to compute the Tg_max_ for the testing set. Overall, the results visualized in Fig. 7 show the similarity of the observed and derived ground temperatures at different soil depths at gages near the three lat/lon profiles. The difference in temperature between the two profiles (Difference = Observation – Derivation, right hand side plots in Fig. 7) is within a relatively narrow range (−3 °C to 3°Celsius), thereby further proving the efficacy of the LSTM model in data reconstruction.

It is clear that reconstruction of Sd is more challenging than Ta and Tg, due to its susceptibility to various local factors and uncertainties^49^, not captured by the input set of predictive variables. Topographical and land cover attributes directly modulate snowpack conditions^89,90^. Moreover, snow depth demonstrates intrinsic seasonality, only measurable during a finite winter period. Consequently, the reliability of the reconstructed snow depth hinges considerably on ERA5-Land skill to accurately capture not only the magnitude, but also the transient snow cover conditions. To partially mitigate this limitation, we implemented “day of year” as an input sequence to inform the LSTM when seasonal snow presence and absence typically manifests at a location. As the results depicted in Supplementary Fig. 3, the trained machine learning model demonstrates skillful estimates of both snow depth and the rate of its increase/decay during the accumulation/melting periods.

However, when the training data are insufficiently long, include predominantly no-snow periods, or there are substantial discrepancies in snow cover durations between the reanalysis product and observations, LSTM performance deteriorates, especially with respect to the reconstructed season length. For example, as shown in Supplementary Fig. 3g, despite the overall reasonable aggregate estimates, the number of snow-absent days of reconstructed data appreciably diverges from the observations. The results indicate that approximately 37% of Sd stations exhibit R^2^ less than 0.5 (Fig. 4b and Supplementary Fig. 4c,d). The skill of reconstructing maximum snow depths is particularly low for the North American and Asia regions, with an average R^2^ of only 0.363 and 0.34 (Fig. 6b-3, 6d-3). Therefore, to promote transparency and inform usage of the reconstructed variables, we provide location-specific model evaluation data along with the statistical performance metrics (i.e., R^2^, KGE, RMSE, and ME) in the publicly shared dataset to inform end-users on reliability of the shared data, as relevant to their specific application.

An important consideration when using ERA5-Land product for data reconstruction is the scale discrepancy of the reanalysis pixels, covering approximately one hundred squared kilometers, relative to the point-level snow depth measurements at stations. ERA5 provides a seamless spatiotemporal grid by assimilating observations and modeling results, and any grid value intrinsically aggregates and averages conditions across a heterogeneous landscape. In regions exhibiting relatively homogeneous meteorological fields and terrain, ERA5-Land cells may reasonably represent the broader area and favorably align with station-based observations. However, in domains characterized by pronounced microclimatological heterogeneities from factors such as complex terrain or land cover, the correlation between ERA5-Land snow variables and *in situ* observations can be weak (Supplementary Fig. 5a,b). This implies that ERA5-Land data may not accurately capture local conditions (e.g., Ta, Sd, and Tg) in the surrounding area, leading to inaccurate reconstruction of snow depth. As illustrated in Supplementary Fig. 5c–e, the reconstructed time series exhibit satisfactory performance. Additionally, since unique ML models are trained at each station, geospatial attributes as potentially informative predictors such as topography, land cover, and geology are excluded from model fitting.

An important question arises concerning suitability of the reconstructed datasets for in-depth studies such as trend analysis, particularly in their ability to accurately reflect climate change. This concern stems from the fact that these datasets are generated by LSTM that was trained and constrained based on a finite amount of training data. Fundamentally, the LSTM performance remains contingent on the quality of the ERA5-Land data. Conceptually, the LSTM acts as a bias correction model for the reanalysis data by integrating available observations to refine gridded estimates and derive missing data at specific locations. If the ERA5-Land dataset appropriately captures temporal trend, the appropriately reconstructed time series will also reproduce a similar temporal pattern. To evaluate this statement, the non-parametric Mann-Kendall and Sen’s Slope estimator tests^91^ for trend analysis were deployed for all variables, with p-value < 0.05 denoting significant trend. The results, presented in Fig. 8, show that the reconstructed data lead to positive trends in 91% and 36% locations for Ta and Tg (calculated only at the shallowest soil depth), a signal consistent with warming across much of the Northern Hemisphere^92,93^. Most snow depth series exhibit no discernible directionality, with only 5% of stations indicating declining seasonal maxima. While this preliminary analysis demonstrates a potential for harnessing the continuous data series for climate change assessments, further data analysis studies are needed to explore the scientific potential of the reconstructed dataset.

Additionally, we conducted a comparison of the trend analysis between the raw data from stations with at least 90% data availability and the reconstructed data. A total of 4,826 stations for Ta and 3,010 stations for Sd met this condition and were utilized for the analysis. We found that 4,773 (98.9%) Ta and 2,963 (98.4%) Sd stations exhibited similar trends to those calculated from the reconstructed data. The comparison of slope values, as illustrated in Supplementary Fig. 6, demonstrates that the trend magnitudes (i.e., slopes) between the raw data and reconstructed data are nearly identical, with R^2^ values of 0.99 and 0.97 for Ta and Sd, respectively. These results indicate that the reconstructed data can be effectively employed for trend analysis while still preserving the primary trends observed in the original data series.

While the evaluation results demonstrate potential accuracy of the novel dataset developed in this study, a summary of limitations is warranted. First, the results indicate that the performance of the LSTM model is unstable and varies not only when comparing across continents but also among stations within the same continent. This outcome can be attributed to the distinct local characteristics of these stations, resulting in varying performance of the trained model. These characteristics may include the length of the training data, the degree of non-stationarity of raw data, and the relationship with the ERA5-Land data. For example, as illustrated in Supplementary Fig. 7, the performance of the LSTM is dependent on the length of the time series used to train the model, as models exhibit better performance when trained with longer data series. Second, when the training data for the LSTM model are sparse, short, and do not capture enough extreme events (especially for snow depth), the performance and reliability of the model outputs may become questionable. As testified by the results, up to 37% of the reconstructed snow depth data exhibit poor performance (as shown in Figs. 4b-e, 6b-3, 6d-3). To provide sufficient information on reliability of the series, evaluation metrics (including R^2^, KGE, RMSE, and ME) have been appended to the metadata, so users can make informed judgment about dataset suitability. Third, the reliability of the reconstructed time series depends considerably on the spatial-temporal reliability of ERA5-Land hydrometeorological fields. ERA5-Land integrates observational data through sophisticated data assimilation procedures using advanced Earth system modeling. While literature attests to the high quality of this recent data product for atmospheric and climate applications, especially data reconstruction^51,94–97^, continued benchmarking of ERA5-Land and the reconstructed data requires further analyses, especially in regional studies, given the documented spatial inconsistencies^33,60,98,99^.

### Supplementary information

Supplementary Figures

## Data Availability

The LSTM model was customized using Python with Keras-Tensorflow library (https://github.com/leriomaggio/deep-learning-keras-tensorflow). The customized Python and figure script can be found at https://github.com/vinhngoctran/datareconstruction.
