# Supplementary material for: Daily station-level records of air temperature, snow depth, and ground temperature in the Northern Hemisphere
Source: Sci Data. 2024 Jun 18;11:645. doi: 10.1038/s41597-024-03483-x (PMC11189437; doi:10.1038/s41597-024-03483-x)
Supplement: Supplementary file 1 — Supplementary Figures [file 41597_2024_3483_MOESM1_ESM.pdf]

Supplementary Figures

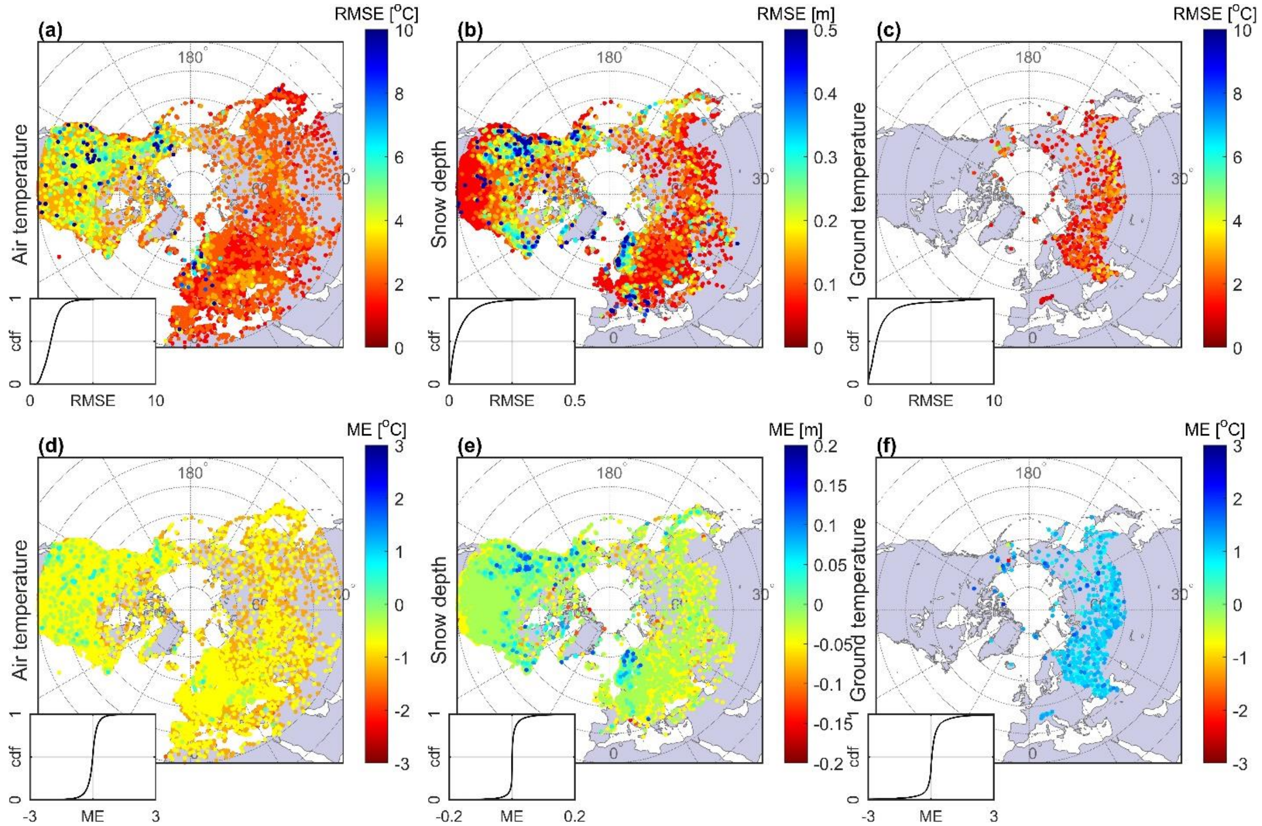

Supplementary Figure 1. Same as Fig. 4, except for RMSE and ME.

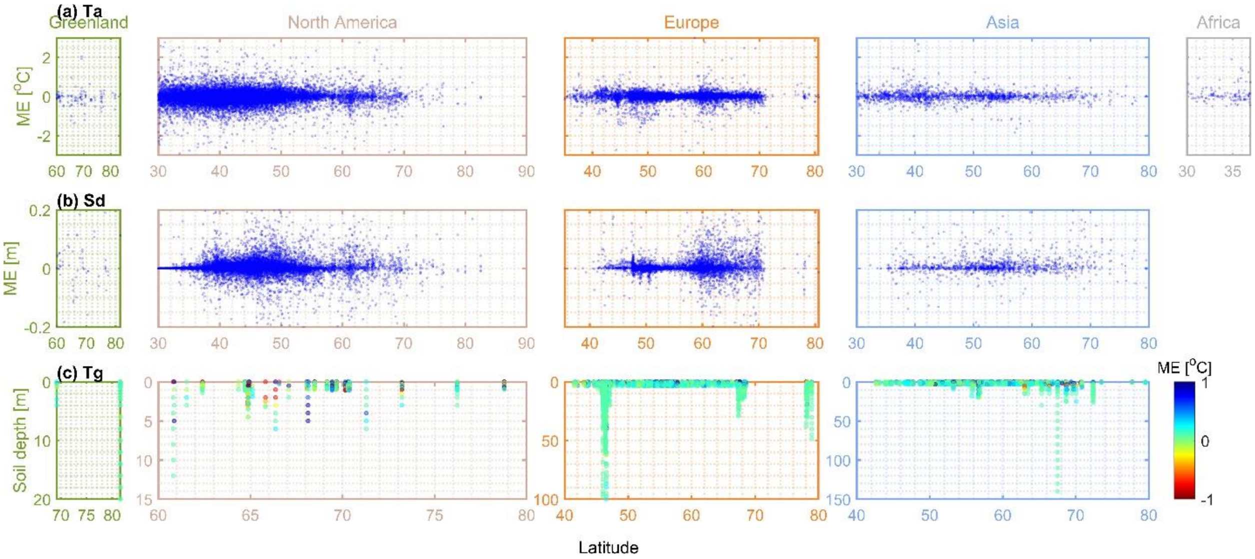

Supplementary Figure 2. Performance of the LSTM for reconstructing Ta, Sd, and Tg based on the mean error (ME) metric. Subplots (a) and (b) respectively present the ME of Ta and Sd, respectively, with the error magnitude denoted on the y-axes. Subplot (c) shows ME for Tg reconstruction at different soil depth. The position of ME dots based on x-axis is plotted based on the latitude of the corresponding station. Positive ME values signify LSTM underestimation relative to observations with reconstructed series lower than measurements and vice versa.

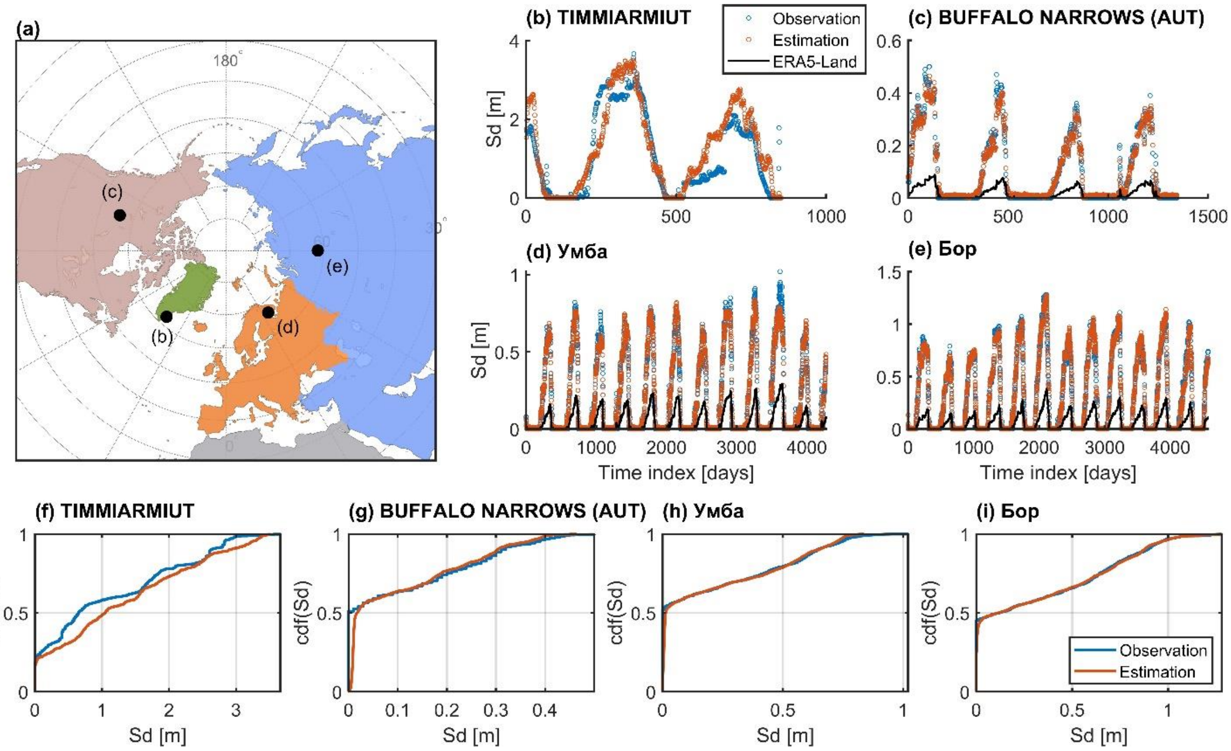

Supplementary Figure 3. LSTM results on snow depth (Sd) reconstruction at exemplary sites. Subplots (b-e) display the observed and reconstructed Sd using LSTM for the locations indicated in (a). Subplots (f-i) show the cumulative distribution function (cdf) of Sd for each station computed over the testing period showed in (b)-(e).

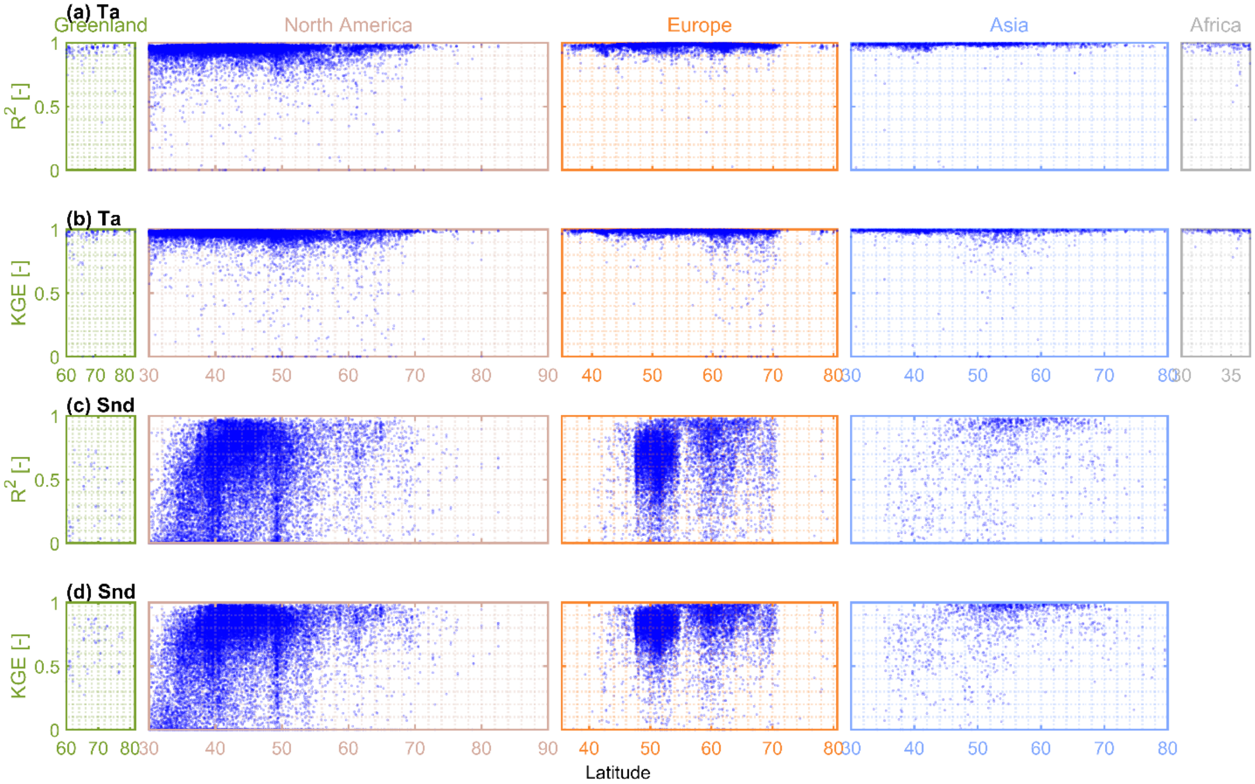

Supplementary Figure 4. Same as Fig. 5, except for Ta and Sd.

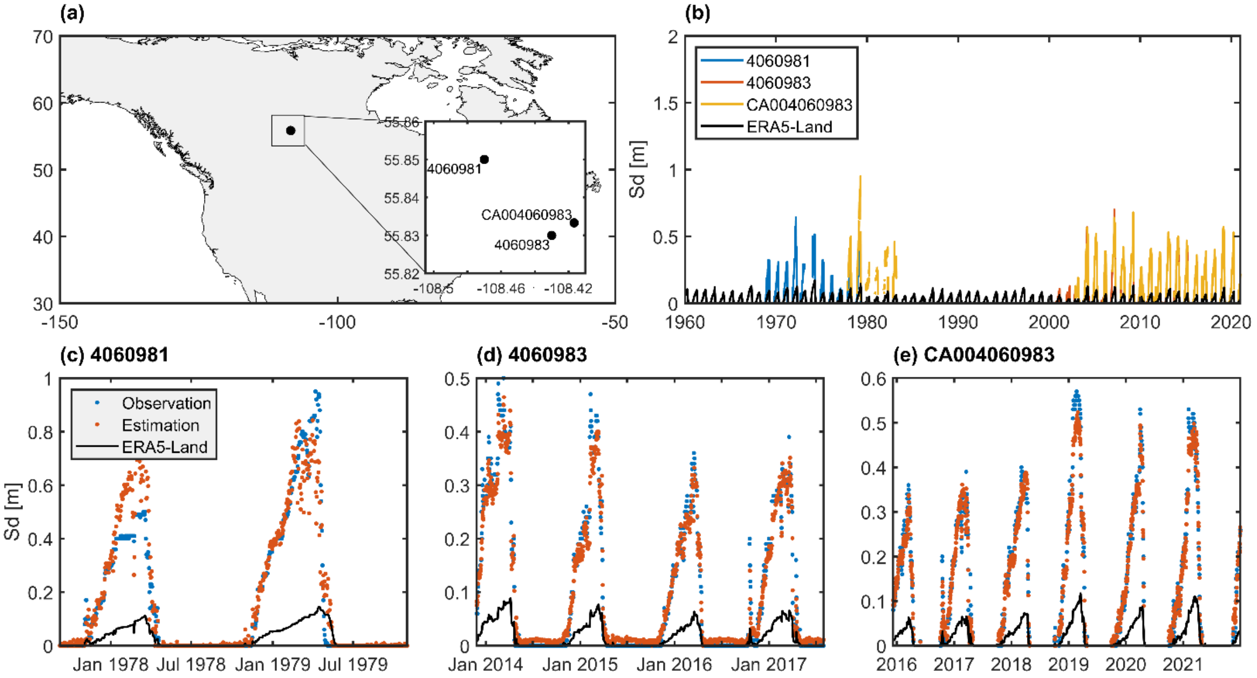

Supplementary Figure 5. Snow depth time series from the ERA5-Land reanalysis data, observations from three stations located within the same ERA5-Land grid cell (0.1° × 0.1°), and the LSTM reconstructed series. Map (a) displays the station locations. Subplot (b) shows the time series of snow depth from the station observations and ERA5-Land from 1960 to 2021. Subplots (c-e) compare the LSTM reconstructed Sd and observations at the stations shown in map (a).

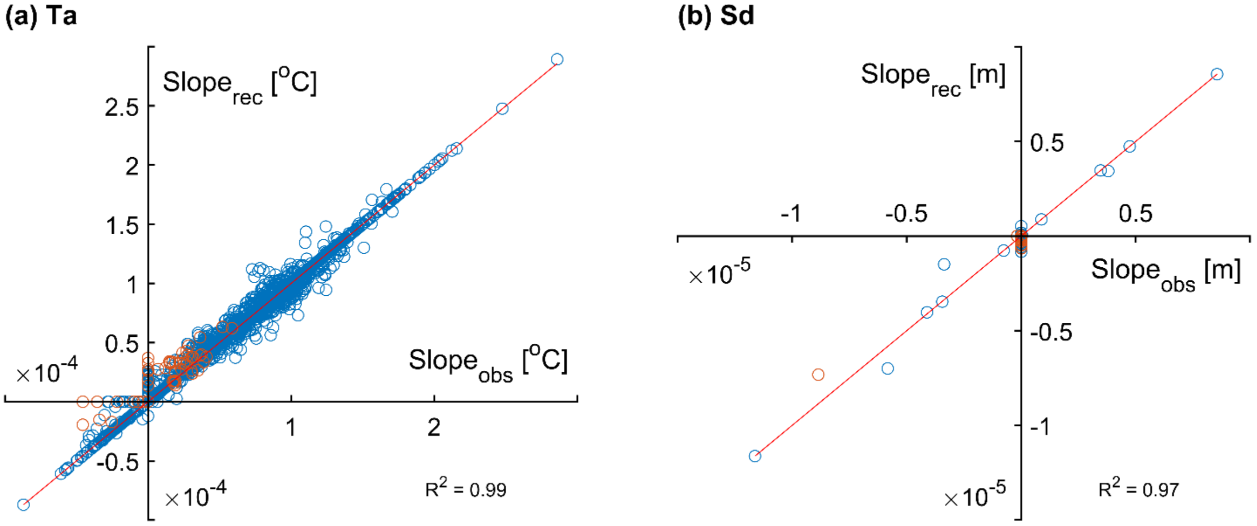

Supplementary Figure 6. Comparison of the trend analysis (based on trend magnitude computed using Sen's slope) between the raw data series ($Slope_{obs}$) and the reconstructed data ($Slope_{rec}$) of Ta and Sd. The calculations were performed for stations with over 90% of recorded data available. The blue circles represent cases where the analysis from the two data series yielded the same trend. The orange circles represent cases where the trend differed between the two data series.

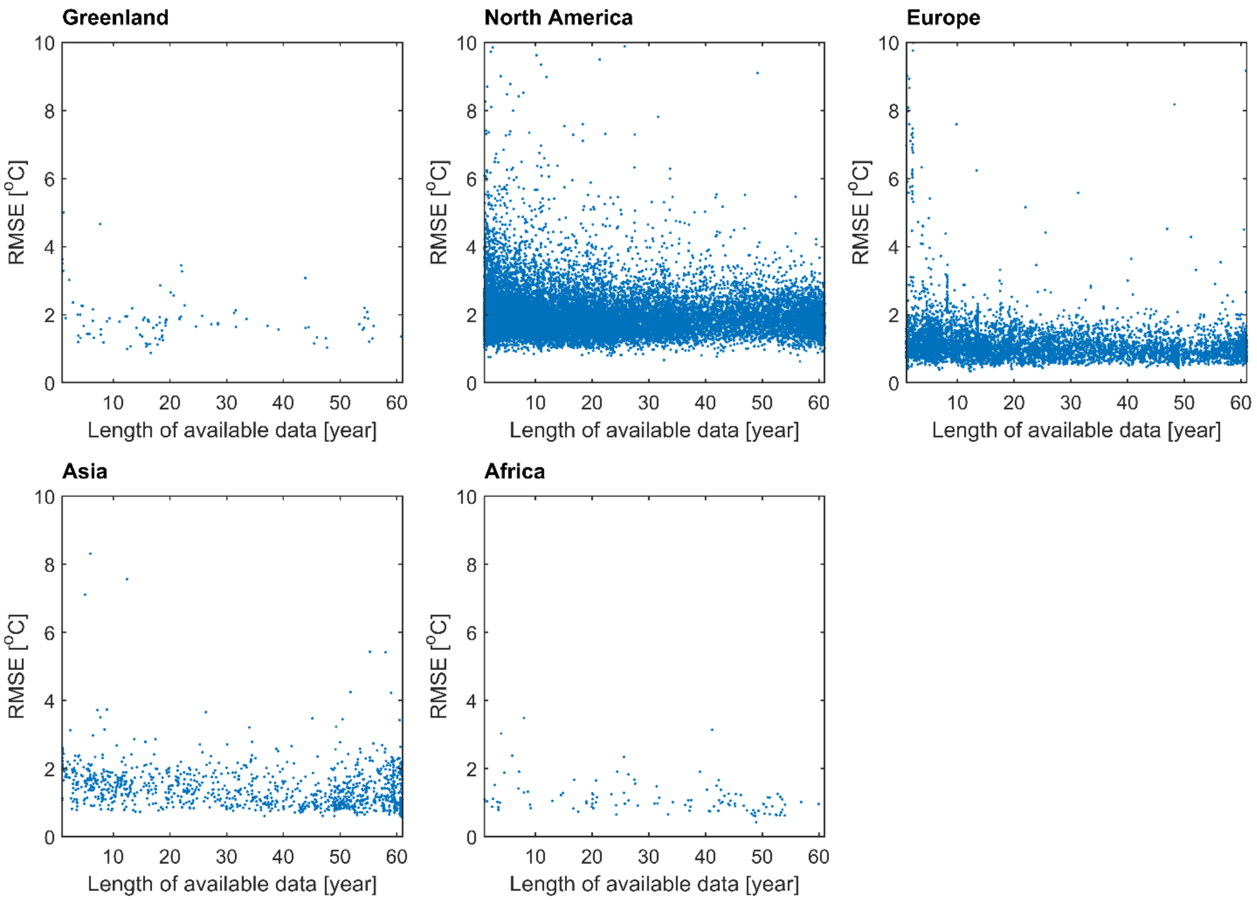

Supplementary Figure 7. Illustrations depicting the relationship between the length of the data record and the performance of the trained model based on root mean square error (RMSE).
